# Selenium-Containing Nano-Micelles Delay the Cellular Senescence of BMSCs Under Oxidative Environment and Maintain Their Regenerative Capacity

**DOI:** 10.3390/bioengineering12090920

**Published:** 2025-08-26

**Authors:** Zirui He, Fangru Xie, Chuanhao Sun, Xuan Wang, Fan Zhang, Yan Zhang, Changsheng Liu, Yuan Yuan

**Affiliations:** 1 The State Key Laboratory of Bioreactor Engineering, East China University of Science and Technology, Shanghai 200237, China; hzr6482@163.com (Z.H.); 17860768813@163.com (X.W.); 2 Frontiers Science Center for Materiobiology and Dynamic Chemistry, East China University of Science and Technology, Shanghai 200237, China; melody999999999@163.com (F.X.); y30230853@mail.ecust.edu.cn (F.Z.); 3 Shanghai Key Laboratory of Advanced Polymeric Materials, Key Laboratory for Ultrafine Materials of Ministry of Education, East China University of Science and Technology, Shanghai 200237, China; cailiaosunch@163.com

**Keywords:** selenium-containing nano-micelles, reactive oxygen species, bone marrow mesenchymal stem cells, cellular senescence

## Abstract

The cellular senescence and functional decline of stem cells are primary contributors to the reduced regenerative capacity and weakened disease resistance in aged tissues. Among the various factors involved, oxidative stress resulting from the accumulation of reactive oxygen species (ROS) is a key driver of stem cell senescence. In an oxidative environment, cells continuously generate ROS, which accelerates cellular senescence and leads to functional deterioration. To intervene in the cellular senescence process of stem cells under such conditions, we selected bone marrow mesenchymal stem cells (BMSCs) as the model system and developed ROS-responsive selenium (Se)-containing nano-micelles capable of efficiently scavenging intracellular ROS. The optimal formulation was determined by modulating the selenium content. Analysis of cellular senescence markers and regenerative capacity reveals that nano-micelles containing 8% Se (Wt %), at a concentration of 15 μg/mL, can significantly modulate ROS levels in BMSCs under oxidative stress, thereby effectively delaying cellular senescence and preserving the osteogenic differentiation potential of BMSCs. These findings offer a promising strategy for mitigating stem cell senescence.

## 1. Introduction

According to the World Health Organization (WHO) report in 2022, the proportion of the global population aged 60 and over is projected to reach 22% by 2050. Age-related impairments in tissue regeneration have emerged as a significant medical challenge, posing serious threats to the health and quality of life of elderly individuals [1,2]. Aging is a prolonged, chronic, dynamic, and multifaceted biological process [3]. Disruption of tissue homeostasis and the diminished regenerative capacity of stem cells are key factors contributing to the reduced ability of aged tissues to regenerate [4]. Therefore, restoring the regenerative capacity of aged tissues by targeting the degraded microenvironment and modulating senescent stem cell function represents a promising therapeutic strategy for age-related diseases [5].

Excessive accumulation of reactive oxygen species (ROS) in the senescent microenvironment is a key driver of stem cells senescence [6]. Mitochondria account for more than 90% of intracellular ROS production and participate in the regulation of cellular signaling through redox-related enzyme systems [7]. Age-associated disruption of mitochondrial homeostasis leads to chronic overproduction of ROS [3,8]. Furthermore, the antioxidant capacities of key enzymes such as NADPH Oxidases (NOXs), superoxide dismutase (SODs), and catalase (CAT) are significantly reduced in senescent cells, thereby amplifying a self-sustaining cycle of ROS accumulation and functional decline [8,9]. The dynamic regulation of redox balance is essential for maintaining immune function and facilitating tissue regeneration [10]. Persistent oxidative stress causes excessive oxidation of critical cellular components, including proteins, lipids, and DNA, which not only undermines cellular integrity but also severely compromises regenerative potential [11,12]. Therefore, timely intervention to modulate the redox environment in aging tissues is crucial for restoring cellular viability and preserving regenerative capacity.

Existing studies have demonstrated that modulating ROS levels and enhancing antioxidant capacity can effectively maintain physiological homeostasis. Currently, major research efforts in antioxidant-based therapeutic strategies focus on small molecule drugs, nano-enzymes that mimic the activity of natural antioxidant enzymes, and functional materials containing ROS-responsive moieties. Specifically, commonly investigated small molecule drugs include N-acetylcysteine (NAC), NAD^+^ and its precursors, resveratrol, curcumin, vitamins, metformin, and polyphenols [13,14,15,16,17,18]. Representative nano-enzyme materials include Fe_2_O_3_ (mimicking CAT activity), CeO_2_ (exhibiting both SOD and CAT activities), and MnO_x_ (mimicking SOD activity) [19,20]. Additionally, functional materials incorporating ROS-responsive components such as thioether, thioketone, disulfide bonds, boronic acid groups, and selenium-containing bonds are also being explored [21,22,23,24]. However, most current studies primarily focus on utilizing antioxidant effects to regulate inflammatory responses and promote tissue regeneration, with limited investigation or discussion on how modulating ROS levels may alleviate cellular senescence and enhance the regenerative capacity of aged tissues [25,26]. Therefore, the development of highly bioactive materials specifically targeting characteristics of senescent cells presents significant potential for clinical translation.

In this study, bone marrow mesenchymal stem cells (BMSCs) exposed to an oxidative environment were employed as the experimental model. Based on selenium (Se), an essential trace element in the human body, an amphiphilic selenium-containing polymer was synthesized [27]. This polymer was capable of self-assembling in aqueous solution to form nano-micelles that could enter BMSCs and dynamically respond to intracellular ROS, thereby delaying cellular senescence while maintaining the osteogenic differentiation capacity of BMSCs. This study further evaluated the impact of varying Se concentrations in nano-micelles on BMSCs’ behavior and proposed a novel strategy to mitigate oxidative stress-induced stem cell senescence, offering new insights into the potential application of stem cells in the treatment of degenerative diseases.

## 2. Materials and Methods

### 2.1. Synthesis of mPEG-b-P(TMC-co-MSeSe) Polymer

The polymerization was conducted under an argon atmosphere using a schlenk line. A diethylene disselenate carbonate dimer (MSeSe) was synthesized according to previous work [28]. Trimethylenecarbonate (TMC, 180 mg, 1.76 mmol), MSeSe (20 mg, 0.36 mmol), and polyethylene glycol monomethyl ether (mPEG_5000_, Mn = 5000, 25 mg, 0.005 mmol) were added into a dry schlenk flask under an argon atmosphere. Lipase CA (20 mg, 10% of monomers) and anhydrous toluene (1.2 mL) were subsequently added to the mixture. The mixture was then stirred at 70 °C for 24 h. After the reaction was completed, the mixture was diluted with 2 mL dichloromethane and filtered. The resulting residue was precipitated in cold anhydrous ether (25 mL) and further dried under vacuum at 40 °C for 24 h to yield a yellow viscous solid, designated as mPEG-*b*-P(TMC-*co*-MSeSe). A series of polymers were synthesized based on the feed ratios presented in Table 1.

### 2.2. Preparation and Characterization of SeSe Nano-Micelles and FITC Loaded SeSe Nano-Micelles

A total of 7 mg of mPEG-*b*-P(TMC-*co*-MSeSe) polymer was dissolved in 3.5 mL of dimethylformamide (DMF). The solution was then added dropwise into 10 mL of stirred ultrapure water and stirred thoroughly for 15 min. Subsequently, the mixture was transferred to a dialysis bag with a molecular weight cutoff of 3500 Da. After 48 h of dialysis using ultrapure water, the resulting micellar solution was diluted to a final concentration of 0.5 mg mL^−1^ for further use. The mPEG-*b*-P(TMC-*co*-MSeSe) polymer was dissolved in DMF according to the proportion described, and an appropriate amount of fluorescein isothiocyanate (FITC) fluorescent dye was added. Then, it was gradually dropped into stirred ultrapure water. Subsequently, the mixture was transferred to a dialysis bag with a molecular weight cut-off of 3500 Da to remove excess DMF and FITC. Thus, FITC-loaded nano-micelles were prepared and then used to analyze the cellular uptake behavior.

### 2.3. Cell Culture in an Oxidative Microenvironment

All experimental animal operating procedures and animal care protocols have been approved by the East China University of Science and Technology (No.: ECUST-2024-048). One-month-old male Sprague-Dawley (SD) rats were euthanized via cervical dislocation and subsequently immersed in alcohol for disinfection over a 15 min period. BMSCs were then isolated from the femurs of the rats. The primary BMSCs were cultured and expanded to the third passage in complete medium supplemented with 10% fetal bovine serum. Subsequently, the cells were induced with aMEM containing 50 μM H_2_O_2_ for 2 h, followed by complete medium containing 10 μM H_2_O_2_. The induced BMSCs were used for subsequent experiments.

### 2.4. Detection of Cell Proliferation

After BMSCs were co-cultured with the nano-micelles, the cells were washed twice with phosphate-buffered saline (PBS) buffer to ensure complete removal of residual culture medium. Subsequently, Alamar Blue working solution was added, and the samples were incubated in the dark for 2 h. Following incubation, the working solution was collected from each well and transferred to a black plate. Fluorescence intensity was then measured at Ex = 520 nm and Em = 590 nm.

### 2.5. Detection of Cellular Uptake of Nano-Micelles

After co-culture of FITC-loaded nano-micelles with H_2_O_2_-induced BMSCs for 4 h, the cells were washed 2–3 times with PBS buffer to ensure that the excess nano-micelles was cleaned. BMSCs were subsequently harvested, and the intracellular fluorescence intensity was detected by flow cytometry (CytoFLEX).

### 2.6. Detection of ROS Levels in BMSCs

The intracellular ROS levels in BMSCs induced by H_2_O_2_ were assessed following 10 h of co-culture with micelles. Intracellular ROS levels were measured by a ROS detection kit (Shanghai, China, Beyotime, S0033) according to the manual provided by the manufacturer. Laser confocal microscopy (Wetzlar, Germany, LEICA TCS SP8) was used to observe the expression of ROS in cells. The fluorescence intensity of cells was analyzed by flow cytometry.

### 2.7. SA-β-gal Staining of BMSCs

After co-culturing H_2_O_2_-induced cells with nano-micelles for 3 days, SA-β-gal staining was performed. Detailed procedural steps followed the manufacturer’s instructions (China, Beyotime, C0602).

### 2.8. Osteogenic Induction of BMSCs

The cells induced by H_2_O_2_ were co-cultured with the osteogenic induction solution (Suzhou, China, OriCell, RAXMX-90021) for 7 or 14 days, and the solution was changed once every 2–3 days.

### 2.9. Detection of ALP Activity in BMSCs

Alkaline phosphatase (ALP) activity was detected after BMSCs were co-cultured with osteogenic induction medium and micelles for 14 days. The co-culture medium was removed, and the cells were washed 2–3 times with PBS buffer to ensure complete removal of residual liquid after each wash. Subsequently, 100 μL of NP-40 cell lysis buffer was added to each well, and the plate was incubated for 30 min at 37 °C to achieve complete cell lysis. The ALP detection working solution was prepared in accordance with the manufacturer’s instructions (China, Beyotime, P0321), ensuring full dissolution and thorough mixing, and was kept on ice prior to use. Then, 50 μL of each sample was transferred into a 96-well plate, followed by the addition of 50 μL of ALP working solution. The mixture was incubated for 15 min at 37 °C. At the end of the incubation period, 100 μL of reaction termination solution was added to each well, and the absorbance was measured at a wavelength of 405 nm.

### 2.10. RNA Analysis Using RT-qPCR

BMSCs were induced with H_2_O_2_ and co-cultured with the material. After 3 days of co-culture, the expression levels of cell senescence-related genes were analyzed. Following 7 days of co-culture in osteogenic induction medium, osteogenesis-related gene expression was assessed. Subsequently, the material was removed, and the cells were rinsed 2–3 times with pre-cooled PBS buffer. The cells were then lysed using pre-cooled Trizol reagent (Tokyo, Japan, TAKARA, 9108), and total RNA was extracted according to standard protocols. RT-qPCR analysis was subsequently carried out using primer sequences listed in Table 2. All primers were purchased from Sangon Biotech Co., LTD (Shanghai, China).

### 2.11. Statistical Analysis

All experimental data are presented as Mean ± SD. Representative results reflect material properties, and cell experiments used five parallel samples. Data processing and statistical analysis were performed using GraphPad Prism software (Version 8.0). One-way ANOVA was used for univariable comparisons across multiple groups, and unpaired two-tailed Student’s *t*-tests were applied for two-group comparisons. Significance levels are defined as follows: * *p* < 0.05, ** *p* < 0.01, *** *p* < 0.005, **** *p* < 0.0001; non-significant differences are marked as “ns” (*p* ≥ 0.05).

## 3. Results and Discussion

### 3.1. Synthesis and Characterization of mPEG-b-P(TMC-co-MSeSe) Polymer

**Figure 1:**
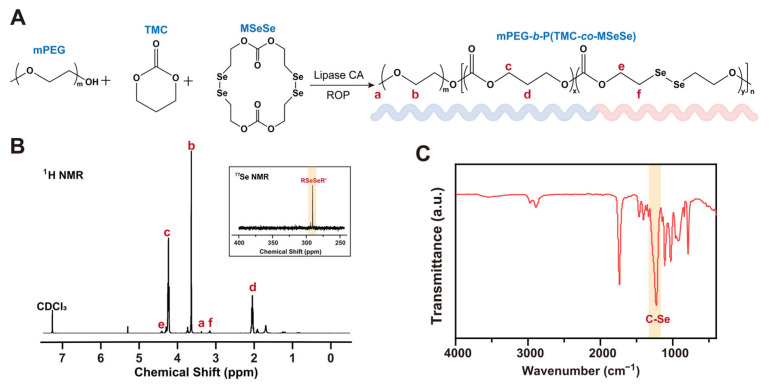
Synthesis and characterization of mPEG-*b*-P(TMC-*co*-MSeSe) polymer. (**A**) Schematic representation of the synthesis of the mPEG-*b*-P(TMC-*co*-MSeSe) polymer. (**B**) ^1^H NMR and ^77^Se NMR spectrogram analysis of the mPEG-*b*-P(TMC-*co*-MSeSe) polymer. (**C**) FTIR spectra of the mPEG-*b*-P(TMC-*co*-MSeSe) polymer in the range of 400–4000 cm^−1^.

Mitochondrial damage induced by oxidative stress exacerbates ROS accumulation [6,11]. Moreover, due to the reduced activity of antioxidant enzymes in senescent cells, their capacity to scavenge ROS becomes inadequate, leading to further deterioration of BMSCs function [29,30]. Therefore, developing a nanomaterial with high ROS responsiveness is crucial for the rapid detection and elimination of intracellular ROS in senescent BMSCs. Selenium (Se) possesses unique redox properties and plays a crucial role in maintaining redox homeostasis within the body [27,31]. In this study, we propose an amphiphilic block copolymer containing Se-Se bonds that exhibits ROS-responsive properties (Figure 1A).

Copolymer mPEG-*b*-P(TMC-*co*-MSeSe) was synthesized by ring-opening polymerization using methoxy polyethylene glycol (mPEG) as the initiator. According to the analysis of the ^1^H NMR spectra (Figure 1B), the signals at 3.38 ppm (a) and 3.65 ppm (b) correspond to protons in the mPEG chain segment. The peaks at 4.24 ppm (c) and 2.05 ppm (d) were attributed to protons in the trimethylene carbonate (TMC) fragment. The peaks located at 4.42 ppm (e) and 3.16 ppm (f) correspond to protons in the OCH_2_CH_2_Se chain segment, respectively. The above results fully confirmed the successful synthesis of mPEG-*b*-P(TMC-*co*-MSeSe). The single peak at 290.50 ppm in the ^77^Se-NMR spectrum confirmed that the Se-Se bond was introduced into the polymer backbone (Figure 1B). As revealed by FTIR analysis (Figure 1C), the asymmetric and symmetric stretching vibrations of the methyl groups on mPEG were observed at approximately 2960 cm^−1^ and 2870 cm^−1^, respectively. A strong and narrow absorption peak near 1740 cm^−1^ corresponds to the C=O stretching vibration of the ester group. Furthermore, a distinct absorption band attributed to the C–Se bond was detected at 1250 cm^−1^. Collectively, these spectral features confirm the successful synthesis of the mPEG-*b*-P(TMC-*co*-MSeSe) polymer.

The molecular weights (*Mn*) of the copolymers were determined by ^1^H NMR spectroscopy and gel permeation chromatography (GPC). In ^1^H NMR analysis, the *Mn* was calculated by comparing the integration of characteristic proton signals from the PMSeSe and PTMC blocks with those of the known protons in the mPEG chain. GPC was employed to measure the relative molecular weights and polydispersity indices. By varying the monomer feed ratios, four different copolymers were prepared. Their *Mn* and key characteristics were listed in Table 3. The discrepancy in the *Mn* between GPC and ^1^H NMR analyses was attributed to difference in hydrodynamic volume between the synthesized copolymers and the polystyrene standards. The polymers self-assembled into nano-micelles in the aqueous phase. The selenium (Se) content in each sample was determined by energy-dispersive X-ray spectroscopy (EDX), and the nano-micelles were subsequently named based on their Se content for further investigation (Table 3). The names of the Se-containing nano-micelles in each group are listed in Table 3.

### 3.2. Characterization of NMSe Nano-Micelles

**Figure 2:**
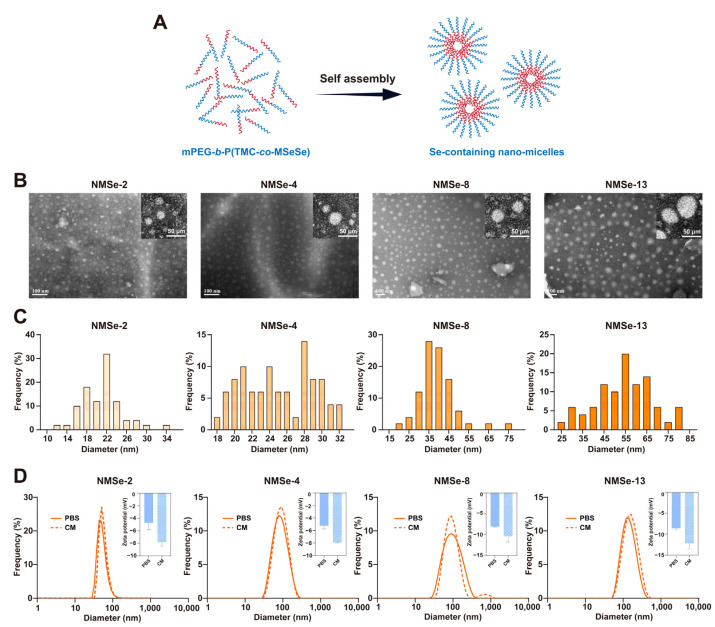
Preparation and characterization of nano-micelles. (**A**) The mPEG-b-P(TMC-c*o*-MSeSe) polymer self-assemble in the aqueous phase to form nano-micelles. The red chain represents the hydrophobic segment of the polymer, whereas the blue chain represents the hydrophilic segment. (**B**) Representative TEM images of NMSe nano-micelles, and the insert is the high magnitude image of nano-micelles. (**C**) Particle size frequency of NMSe nano-micelles. (**D**) The particle size and zeta potential of the nano-micelles were measured in both phosphate-buffered saline (PBS) and culture medium (CM) using dynamic light scattering (DLS).

To promptly respond to intracellular ROS, mPEG-*b*-P(TMC-*co*-MSeSe) amphiphilic polymers self-assemble in aqueous solution to form Se-containing nano-micelles (NMSe) (Figure 2A). The designations of nano-micelles are summarized in Table 3. A Transmission Electron Microscope (TEM) was employed to examine the morphology of the nano-micelles in each group. As shown in Figure 2B, nano-micelles with uniform size and an excellent dispersion state were formed across all groups.

The size of nanomaterials is a critical factor influencing their cellular uptake and subsequent functional effects [32]. The particle size distribution of nano-micelles in each group was counted (Figure 2C). The size of the nano-micelles was 21.12 ± 4.05 nm in the NMSe-2 group, 25.05 ± 3.96 nm in the NMSe-4 group, 39.30 ± 9.40 nm in the NMSe-8 group, and 54.17 ± 13.11 nm in the NMSe-13 group. To further investigate the behavior of nano-micelles in cell culture systems, the nano-micelles were dispersed in both PBS buffer and serum-containing culture medium (CM), followed by measurements of particle size distribution and zeta potential by dynamic light scattering (DLS) (Figure 2D). The size of the nano-micelles in PBS buffer and serum-containing CM were 84.84 ± 28.62 nm and 93.30 ± 34.03 nm in the NMSe-2 group, 90.13 ± 41.41 nm and 95.62 ± 38.20 nm in the NMSe-4 group, 105.40 ± 63.51 nm and 106.00 ± 87.50 nm in the NMSe-8 group, and 135.05 ± 87.83 nm and 165.59 ± 104.61 nm in the NMSe-13 group. These data indicate that all the NMSe nano-micelles have the potential to be endocytosed into cells [32]. The Zeta potentials of the nano-micelles in PBS buffer and CM were −4.72 ± 1.12 mV and −7.78 ± 0.67 mV in the NMSe-2 group, −5.24 ± 0.53 mV and −7.91 ± 0.07 mV in the NMSe-4 group, −8.05 ± 0.11 mV and −10.38 ± 1.40 mV in the NMSe-8 group, and −8.58 ± 0.45 mV and −12.17 ± 1.41 mV in the NMSe-13 group.

### 3.3. NMSe Regulates Intracellular ROS Levels in BMSCs in Oxidative Microenvironment

**Figure 3:**
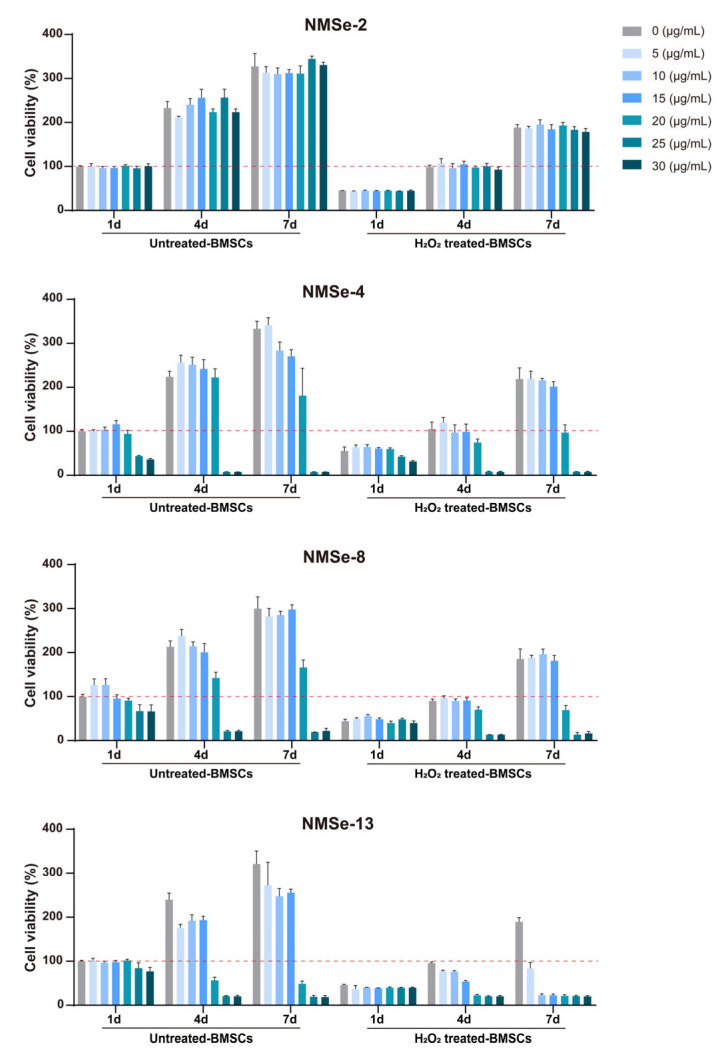
Biocompatibility analysis of NMSe nano-micelles. The proliferation of BMSCs co-cultured with NMSe nano-micelles at varying concentrations. The red dotted line represents the cell survival rate level of the control group at 1 day.

To further investigate the effect of NMSe on BMSCs viability, the proliferation of BMSCs co-cultured with varying concentrations of NMSe was assessed. H_2_O_2_-treated BMSCs were used to simulate BMSCs under oxidative stress. As the Se content increased, the proliferation ability of BMSCs gradually decreased, particularly in the cells treated with H_2_O_2_ (Figure 3). One reason is that Se-Se reactions exhibit high efficiency under oxidative conditions, leading to the formation of SeOOH, a compound that demonstrates greater biological toxicity [33,34]. To ensure cell viability, a nano-micelle concentration of 15 μg/mL was selected for all subsequent cell experiments.

**Figure 4:**
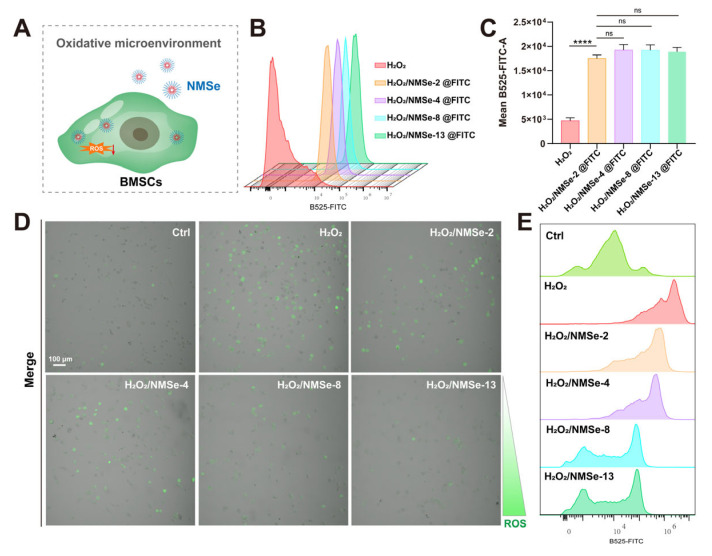
NMSe regulates intracellular ROS levels in BMSCs in oxidative microenvironment. (**A**) NMSe nano-micelles are internalized by BMSCs in response to intracellular ROS. (**B**) The cellular uptake of FITC-loaded NMSe nano-micelles by BMSCs was assessed using flow cytometry. (**C**) Quantitative analysis of the mean intracellular fluorescence intensity of BMSCs after uptake of FITC-loaded nano-micelles. (**D**) The intracellular ROS levels in BMSCs were assessed using confocal microscopy. (**E**) The intracellular ROS levels in BMSCs were assessed using flow cytometry. (**** *p* < 0.0001; ns *p* ≥ 0.05).

NMSe nano-micelles were developed as a strategy to modulate intracellular ROS levels (Figure 4A). To investigate the uptake of NMSe nano-micelles by BMSCs in an oxidative environment, BMSCs treated with H_2_O_2_ were co-cultured with fluorescent NMSe nano-micelles loaded with FITC, and the intracellular fluorescence intensity was detected. All NMSe nano-micelle groups could be efficiently internalized by BMSCs in the H_2_O_2_-treated group (Figure 4B). The average intracellular fluorescence intensity of BMSCs internalizing FITC-labeled nano-micelles remained comparable across groups under an oxidative microenvironment (Figure 4C). The intracellular ROS levels in BMSCs were further detected using the DCFH-DA probe to evaluate the regulatory effects of NMSe nano-micelles. The intracellular ROS level in BMSCs was markedly elevated in the H_2_O_2_ group compared to that in the Ctrl group. Following treatment with NMSe nano-micelles, the ROS level in H_2_O_2_-treated BMSCs was effectively reduced (Figure 4D). Flow cytometry was further utilized to analyze the intracellular ROS distribution patterns in BMSCs following treatment with NMSe nano-micelles (Figure 4E). The Se content influences the capacity of NMSe nano-micelles to regulate intracellular ROS, with higher selenium content correlating with enhanced regulatory. Specifically, the intracellular ROS levels in H_2_O_2_-treated BMSCs co-cultured with NMSe-8 and NMSe-13 were comparable to those in the Ctrl group.

### 3.4. NMSe Nano-Micelles Delay BMSCs Senescence in Oxidative Microenvironment

**Figure 5:**
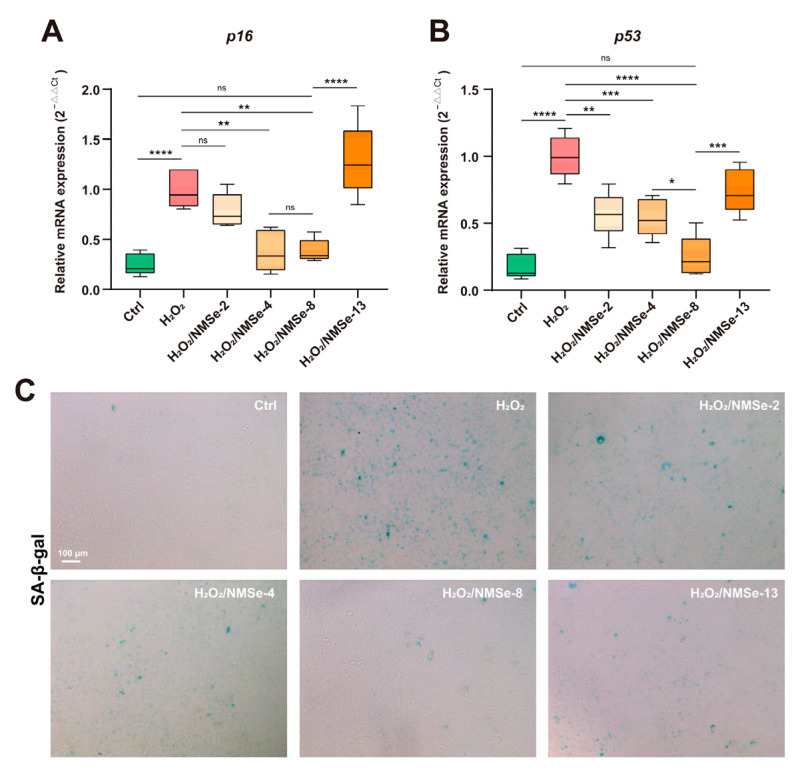
Expression of senescence-related genes in BMSCs. (**A**) RT-qPCR analysis of *p16* in BMSCs. (**B**) RT-qPCR analysis of *p53* in BMSCs. (**C**) SA-β-gal staining of BMSCs. (* *p* < 0.05, ** *p* < 0.01, *** *p* < 0.005, **** *p* < 0.0001; ns *p* ≥ 0.05).

The intracellular ROS levels in BMSCs under an oxidative environment were modulated by NMSe nano-micelles. This study further investigated their regulatory impact on the cellular senescence of BMSCs under oxidative stress. *p16* and *p53* are crucial regulators in maintaining cellular function and homeostasis, playing significant roles in cell proliferation, tumorigenesis, and senescence [35]. In this study, the expression levels of *p16* and *p53* were quantified using RT-qPCR. The results indicated that BMSCs in an oxidative environment showed significantly high expression of *p16* and *p53,* suggesting that oxidative stress accelerated the cellular senescence of BMSCs. The H_2_O_2_/NMSe-8 group exhibited the most pronounced effect in modulating the senescence status of BMSCs under oxidative conditions. Compared with the H_2_O_2_-treated BMSCs, co-culturing with NMSe-8 resulted in a 61.4 ± 11.4% reduction in *p16* expression and a 75.2 ± 15.5% reduction in *p53* expression. To further verify the effect of nano-micelles on the senescence level of BMSCs under oxidative stress, H_2_O_2_-treated BMSCs co-cultured with different groups of nano-micelles were analyzed using SA-β-gal staining. As shown in Figure 5C, H_2_O_2_ treatment markedly increased the number of SA-β-gal-positive cells in BMSCs. However, co-culturing with NMSe nano-micelles significantly reduced SA-β-gal expression, particularly in the NMSe-8 group. These data indicate that NMSe-8 nano-micelles rapidly reduce intracellular ROS levels in BMSCs under oxidative stress, thereby contributing to the delay of BMSCs senescence.

### 3.5. NMSe Nano-Micelles Maintain the Osteogenic Ability of BMSCs in Oxidative Microenvironment

**Figure 6:**
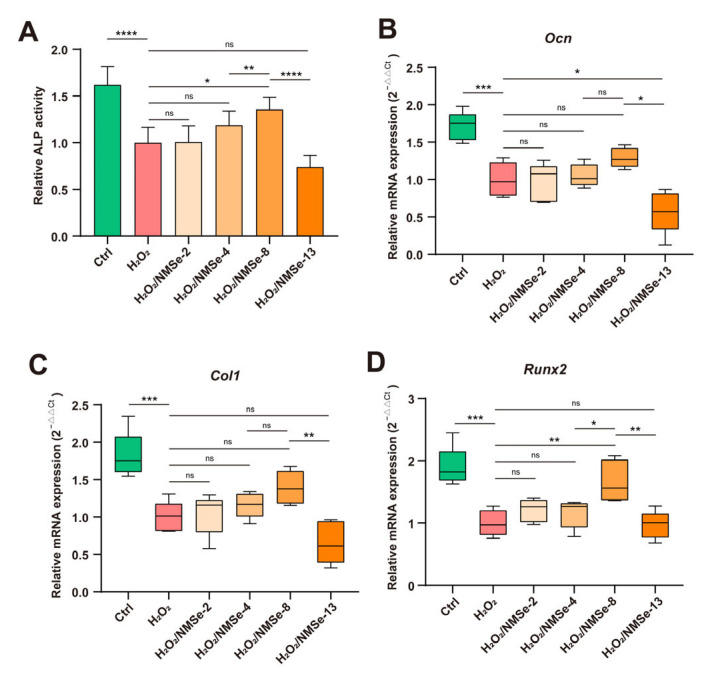
Characterization of osteogenic properties of BMSCs. (**A**) Quantification of ALP activity in BMSCs on Day 14. (**B**) RT-qPCR analysis of *Ocn* in BMSCs on Day 7. (**C**) RT-qPCR analysis of *Col1* in BMSCs on Day 7. (**D**) RT-qPCR analysis of *Runx2* in BMSCs on Day 7. (* *p* < 0.05, ** *p* < 0.01, *** *p* < 0.005, **** *p* < 0.0001; ns *p* ≥ 0.05).

The regenerative capacity of stem cells plays a crucial role in maintaining tissue homeostasis and determining the effectiveness of treatments for degenerative diseases [4]. To further evaluate the impact of NMSe on intracellular ROS level regulation and its subsequent influence on the osteogenic regeneration capacity of BMSCs, ALP activity and the expression levels of osteogenesis-related genes were analyzed. During the process of osteogenic induction, elevated oxidative stress levels within the microenvironment can impede the osteogenic differentiation capacity of BMSCs. Compared with the H_2_O_2_ group, the ALP activity of BMSCs co-cultured with NMSe increased by 35.6 ± 13.2% (Figure 6A). The markers of bone formation were further assessed. Compared with the H_2_O_2_ group, the H_2_O_2_/NMSe-8 group exhibited increased expression levels of *Ocn*, *Col1*, and *Runx2* by 29.1 ± 13.3%, 39.2 ± 22.2%, and 66.7 ± 33.4%, respectively (Figure 6B–D). These results suggest that NMSe nano-micelles preserved the osteogenic differentiation capacity of BMSCs under oxidative stress conditions.

## 4. Conclusions

The excessive accumulation of ROS and the subsequent oxidative stress response are key factors leading to stem cell senescence and the impairment of regenerative capacity in aged tissues. This study aims to precisely and comprehensively regulate the ROS levels within BMSCs under oxidative conditions. Here, ROS-sensitive selenium-containing polymers were designed, which can self-assemble into nano-micelles, enter cells, and effectively scavenge intracellular ROS. This study further explored the effects of nano-micelles with different Se contents on delaying the oxidative senescence of BMSCs and maintaining their regenerative capacity. Among them, nano-micelles containing 8% selenium (Wt %) can significantly regulate the intracellular ROS levels in BMSCs under oxidative conditions, bringing them close to those observed in the control group. In an oxidative microenvironment, NMSe-8 nano-micelles markedly reduced the expression of senescence-related markers in BMSCs, such as *p16* by approximately 61.4% and *p53* by approximately 75.2%, indicating their potential to delay cellular senescence and thereby help maintain the cells’ osteogenic differentiation capacity. This strategy offers a promising therapeutic approach for addressing age-related oxidative stress in stem cells.

## Figures and Tables

**Table 1:** Feeding rate and nomenclature.

mPEG_5000_ (mg)	TMC (mg)	MSeSe (mg)	Nomenclature of Polymers
25	195	5	PSe5
25	190	10	PSe10
25	180	20	PSe20
25	170	30	PSe30

**Table 2:** RT-qPCR primer sequences.

Target Gene	Forward Primer Sequence (5′-3′)	Reverse Primer Sequence (5′-3′)
*Gapdh*	TGACCACAGTCCATGCCATC	GACGGACACATTGGGGGTAG
*p16*	CTCCTTGGCTTCACTTCTGG	CTCCCTCCCTCTGCTAACCT
*p53*	CTACTTCCCAGCAGGGTGTC	CAGACCAAGAGGTTGGGTCG
*Col1*	TGGATGGCTGCACGAGT	TTGGGATGGAGGGAGTTTA
*Runx2*	ATCCAGCCACCTTCACTTACACC	GGGACCATTGGGAACTGATAGG
*Ocn*	GCCCTGACTGCATTCTGCCTCT	TCACCACCTTACTGCCCTCCTG

**Table 3:** Characterization of polymers and nano-micelles.

Polymer	Mn ^a^ (KDa)	Mn ^b^ (KDa)	PDI	Se Content in Nano-Micelles (Wt %)	Nomenclature of Nano-Micelles
PSe5	20.9	15.7	1.28	1.9	NMSe-2
PSe10	21.0	15.9	1.35	4.4	NMSe-4
PSe20	21.7	16.4	1.36	8.1	NMSe-8
PSe30	22.2	16.5	1.42	13.4	NMSe-13

^a^ Molecular weight determined by ^1^H NMR; ^b^as determined by GPC.

## Data Availability

Data are contained within this article.

## Abbreviations

The following abbreviations are used in this manuscript:

ROS	Reactive oxygen species
BMSCs	Bone marrow mesenchymal stem cells
Se	Selenium
mPEG	Polyethylene glycol monomethyl ether
TMC	Trimethylenecarbonate
MSeSe	Diethylene disselenate carbonate dimer
PSe	Polymer of mPEG-*b*-P(TMC-*co*-MSeSe)
NMSe	Se-containing nano-micelles
PBS	Phosphate-buffered saline
CM	Culture medium
Alp	Alkaline phosphatase
RT-qPCR	Real-time quantitative polymerase chain reaction
